# ECCO2R and NIV-NAVA for stepwise early weaning in extremely severe COPD patients: a promising solution with details to be defined

**DOI:** 10.1186/s13054-020-2735-8

**Published:** 2020-01-28

**Authors:** Heyan Wang, Hangyong He

**Affiliations:** 1 Department of Critical Care Medicine, The Sixth Hospital of Guiyang, Guiyang City, Guizhou Province China; 2 Department of Respiratory and Critical Care Medicine, Beijing Institute of Respiratory Medicine, Beijing Chao-Yang Hospital, Capital Medical University, No. 8 Gongren Tiyuchang Nanlu, Chaoyang District, Beijing, 100020 China

Dear editor,

We read with great interest of the report by Karagiannidis and colleagues [1] about the usefulness of the electrical activity of the diaphragm (Edi) signal to monitor and guide patients with severe acute exacerbation of chronic obstructive pulmonary disease (COPD) on veno-venous extracorporeal CO_2_ removal (ECCO_2_R) and neurally adjusted ventilatory assist non-invasive ventilation (NIV-NAVA). They found the Edi during ECCO_2_R weaning was the best predictor of tolerance to removing ECCO_2_R. Interestingly, based on their study, a stepwise weaning protocol for extremely severe COPD is worth being proposed. Their first step was rapid weaning from invasive mechanical ventilation (IMV) by correcting respiratory acidosis with ECCO_2_R. Then they used NIV-NAVA to give patients partial respiratory support after extubation. The final step was further weaning from ECCO_2_R guided by Edi prediction. And they used this strategy to get successful early extubation in 20 patients, 12 early weaning from ECCO_2_R, and 19 patients (95%) were finally weaned from ECCO_2_R and discharged home. However, some details in the use of ECCO_2_R and NIV-NAVA are needed to be further clarified.

First, in Karagiannidis’s study [1], the investigators applied an average ECCO_2_R blood flow (BF) of 2.1 L/min in the initial phase, which is higher than what is typically applied in COPD exacerbations. This may suggest that they actually used a mini-ECMO for both oxygenation support and CO_2_ removal in getting an early stabilization and weaning from IMV, rather than the classic ECCO_2_R with low BF of 1 L/min or less reported in other studies [2]. And the occurrence of progressive hypoxemia has been reported in COPD patients treated by ECCO_2_R, which may need extra oxygenation support with a high BF [3]. Thus, it is still unclear which level of BF and its counterpart extracorporeal support system (ECCO_2_R or mini-ECMO) is more suitable for early weaning in extremely severe COPD.

Second, five major bleeding events occurred during ECCO_2_R in Karagiannidis’s report [1], which prolonged ICU length of stay but had no impact on mortality. Therefore, although rapid extubation with ECCO_2_R would be preferred, such a strategy has to be weighed against the potentially severe complications of ECCO_2_R [4].

Third, the features of the ECCO_2_R circuit are also of crucial importance in determining treatment success. The dual-lumen cannula access facilitated early rehabilitation [5]. However, Karagiannidis and colleagues [1] did not use a dual-lumen cannula for ECCO_2_R to get an early ambulation. Therefore, dual-lumen cannula may be a factor which may help in avoiding delayed recovery and weaning failure.

Finally, ineffective triggering during NIV is commonly seen in severe COPD patients with high intrinsic PEEP and could lead to NIV failure. NAVA is especially helpful in avoiding ineffective triggering and unloading the patients with good synchronization. Thus, Edi by NIV-NAVA is not only a predictive factor for ECCO_2_R weaning, but also provided better respiratory support during and after ECCO_2_R weaning.

Overall, for investigating the stepwise early weaning strategy of extremely severe COPD from IMV, it will be important to define details about the right requirement of BF and selection of cannula for ECCO_2_R and using NIV-NAVA both for better NIV synchronization and prediction of ECCO_2_R weaning.

## Data Availability

Not applicable
